# Nervousness

**Published:** 1860-10

**Authors:** 


### NERVOUSNESS.

It is certainly satisfactory to know that there are persons in different parts of the country who have sent for the various publications of the Editor, and who make it a work of love to their kind to distribute them among their neighbors, and give them to strangers, as a means of leading them to health. A wealthy Carolina planter, who claims to have "one of the best wives in the world," writes: "I loaned 'Consumption' to a traveller who was threatened with it; and 'Health and Disease' to a minister, and the six bound volumes of the JOURNAL OF HEALTH have gone on errands of doing good." He applied to us for medical advice some time ago. He was full of the fidgets; was a bundle of nerves, every one of which had some complaint to make every now and then; at another time they would all squall out together, then he would literally faint away; at other times he felt an insupportable "goneness" at the stomach, and often wished it had "gone," for there was such an incessant "gnawing" especially before dinner, that he felt as if he must eat something or die. We sent him some medicine and advised him to die, or at least to make the experiment to see whether it would kill him or not, rather than be such a slave to his "belly." At an interval of some months he sends us—not our fee, that we always take before we give advice, for then we know that we are paid, and work cheerfully and hopefully. We medicate by the month, not by the job, because we want to make our patients spry and improve their time, and not hang on our hands indefinitely and run up long bills against themselves. If they don't begin to get decidedly better within a month, it is a "sign" that they would do well to go elsewhere. As we were saying, our quondam patient writes that he has not had as good health in seven years, and that he attributes it entirely to our advice. Some body began to sniff a mice just then; "entirely to your advice!" He took every thing—but our pills. We thought of publishing the letter until we came to that part of it inquiring, "Will they keep good until next summer?" This is "July 30, '60," and the pills were sent last April! If he had only left out that part of it, what a good "certificate" we would have had!

There are, however, several valuable lessons to be drawn by our readers from this narration. FIRST: Serious ailments may be cured without physic. SECOND: Yielding to the gnawings of the stomach before meal-times is generally a means of fixing the dyspepsia. THIRD: A judicious system of dieting, that is, eating plain, nourishing food at regular times and in moderate amounts, is sometimes happily efficacious in removing that "nervousness," or "nervous irritability," which not only makes the life of the dyspeptic or the bilious wretched, but makes the members of their families more or less so. The subject certainly merits the consideration of nervous persons.

Nervousness and dyspepsia may be and are generally cured without starvation or medicine; in fact, they are often aggravated thereby. Dieting, starving, is good in its place, but it has been unwisely practiced in many cases, and life has paid the forfeit. Exercise suitably conducted is an important means of invigoration; but taken injudiciously, it kills rather than cures. But how to order the exercise and how to appoint the food in quantity, quality, and frequency, when to give medicine and when to withhold it, to the surest benefit and highest safety of the suffering, requires the learning, the experience, the observation, and the comparison of a lifetime. Yet millions daily give and take medical advice from one single experience or observation, and multitudes daily die in consequence.

